# *Trans*-scirpusin A showed antitumor effects via autophagy activation and apoptosis induction of colorectal cancer cells

**DOI:** 10.18632/oncotarget.17388

**Published:** 2017-04-24

**Authors:** Eun-Hye Hong, Eun-Young Heo, Jae-Hyoung Song, Bo-Eun Kwon, Jae-Young Lee, Yaejeong Park, Jinwoong Kim, Sun-Young Chang, Young-Won Chin, Sang-Min Jeon, Hyun-Jeong Ko

**Affiliations:** ^1^ Laboratory of Microbiology and Immunology, College of Pharmacy, Kangwon National University, Chuncheon, Gangwon-do 24341, Korea; ^2^ Laboratory of Microbiology, College of Pharmacy, and Research Institute of Pharmaceutical Science and Technology (RIPST), Ajou University, Suwon, Gyeonggi-do 16499, Korea; ^3^ Lab of Cancer Signaling and Metabolism Network, College of Pharmacy, and Research Institute of Pharmaceutical Science and Technology (RIPST), Ajou University, Suwon, Gyeonggi-do 16499, Korea; ^4^ College of Pharmacy and Research Institute of Pharmaceutical Sciences, Seoul National University, Seoul, 08826, Korea; ^5^ College of Pharmacy and Integrated Research Institute for Drug Development, Dongguk University-Seoul, Goyang, Gyeonggi-do 10326, Korea; ^6^ Convergence Research Center for Functional Plant Products, Advanced Institutes of Convergence Technology, Yeongtong-gu, Suwon, Gyeonggi-do 16229, Korea

**Keywords:** trans-scirpusin A, colorectal cancer cells, AMPK, autophagy, apoptosis

## Abstract

*Trans*-Scirpusin A (TSA) is a resveratrol oligomer found in *Borassus flabellifer L*. We found that TSA inhibited the growth of colorectal cancer Her2/CT26 cells *in vivo* in mice. Although some cytotoxic T lymphocytes (CTLs) were induced against the tumor-associated antigen Her2, TSA treatment did not significantly increase the level of Her2-specific CTL response compared to that with vehicle treatment. However, there was a significant increase in the level of TNF-α mRNA in tumor tissue and Her2-specific Ab (antibody) production. More importantly, we found that TSA overcomes the tumor-associated immunosuppressive microenvironment by reducing the number of CD25^+^FoxP3^+^ regulatory T cells and myeloid-derived suppressor cells (MDSCs). We detected the induction of autophagy in TSA-treated Her2/CT26 cells, based on the increased level of the mammalian autophagy protein LC3 puncta, and increased conversion of LC3-I to LC3-II. Further, TSA induced 5' AMP-activated protein kinase (p-AMPK) (T172) and inhibited mammalian target of rapamycin complex 1 (mTORC1) activity as estimated by phosphorylated ribosomal protein S6 kinase beta-1 (p-p70S6K) levels, thereby suggesting that TSA-mediated AMPK activation and inhibition of mTORC1 pathway might be associated with autophagy induction. TSA also induced apoptosis of Her2/CT26 cells, as inferred by the increased sub-G1 mitotic phases in these cells, Annexin V/PI-double positive results, and TUNEL-positive cells. Finally, we found that the combined treatment of mice with docetaxel and TSA successfully inhibited tumor growth to a greater extent than docetaxel alone. Therefore, we propose the use of TSA for supplementary anticancer therapy to support anti-neoplastic drugs, such as docetaxel, by inducing apoptosis in cancer cells and resulting in the induction of neighborhood anti-cancer immunity.

## INTRODUCTION

Polyphenols are produced as secondary metabolites in plants and are consumed as part of a normal diet by humans [1]. Dietary polyphenols are found in fruits, tea, vegetables, and wine, and are mostly derivatives and isomers of flavonols, catechins and phenolic acid [1]. Polyphenols are documented to have multiple properties including antioxidant [2], anti-inflammatory [3, 4], anti-atherosclerosis [5] and anti-cancer activities [6–8].

Among dietary polyphenols, resveratrol (3,4’,5-trihydroxy-trans-stilbene), found in the skin of grapes, blueberries, raspberries and mulberries [9], is known to be effective against bacterial and fungal infections [10, 11], cardiovascular diseases [12], metabolic diseases [13], and cancer [14–16]. Resveratrol has also been reported to show anti-inflammatory effects [17], inhibit lipid peroxidation [18], and activate free radical scavenging [19], further supporting the evidence in the favor of the antioxidant activity of resveratrol. In addition to the anti-neoplastic effect of resveratrol [20], resveratrol could be used along with irradiation and chemotherapy for cancer therapy [21], thus enabling its supplementary use with chemotherapy in cancer patients.

In this study, we investigated the anti-cancer effect of TSA, a novel resveratrol oligomer isolated from *B. flabellifer L*.. Scirpusin A is a hydroxystilbene dimer from Xinjiang wine grapes with anti-obesity and anti-adipogenic activity, and with inhibitory effects on amyloid-β aggregation [22] and a protective role against DNA damage by singlet oxygen [23]. Scirpusin B is a piceatannol dimer obtained from passion fruit (*Passiflora edulis*) seeds and has vasorelaxing and anti-HIV effects [24]. The biological effect of TSA, however, has not yet been documented, and therefore, we studied whether TSA has anti-cancer activity both *in vitro* and *in vivo*.

## RESULTS

### TSA inhibited the growth of colorectal cancer cells

**Figure 1:**
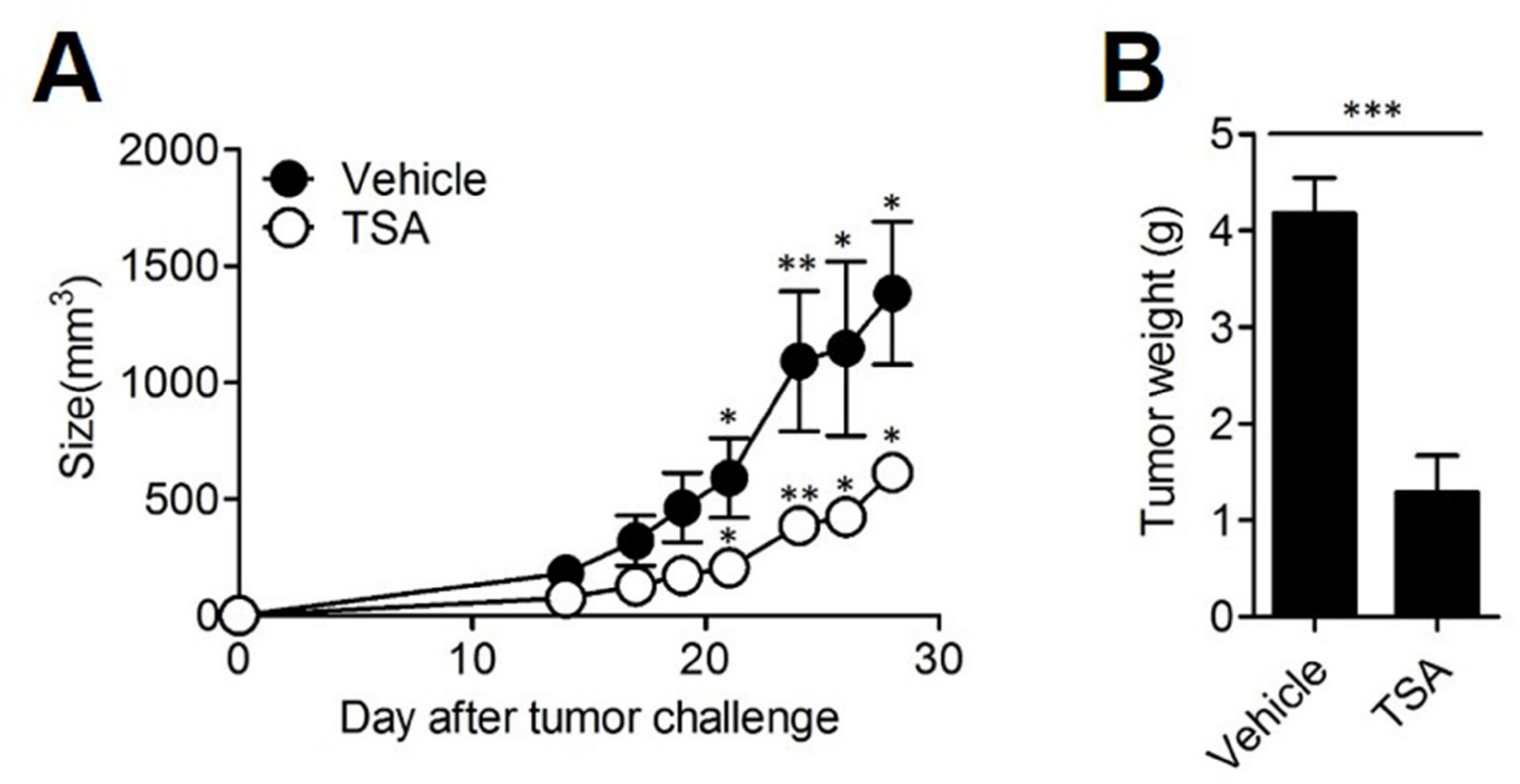
TSA decreased the growth of tumor *in vivo* **(A)** BALB/c mice received s.c. injection of 2×10^5^ Her2/CT26 tumor cells expressing human Her-2/neu. Mice were treated with TSA or vehicle until tumor size reached 50-100 mm^3^ (size of the tumor was measured 3 times per week). Tumor growth was measured (*p<0.05, **p<0.01, Student's *t*-test; n=5 per group). **(B)** Four weeks after TSA administration, mice were sacrificed. Tumor tissue was isolated, and the weight of each tumor was measured (***p<0.001, Student's *t*-test; n=5 per group).

To study the anti-cancer effects of TSA, we assessed the effect of TSA in the syngeneic tumor transplant model of Her2/CT26 cells in which human Her-2/neu (Her2) was the tumor-associated antigen. In mice with the Her2/CT26 transplant, administration of TSA delayed tumor growth compared to that in mice treated with a vehicle (Figure 1A). Similarly, tumor weight significantly decreased when TSA treatment was given to mice (Figure 1B).

**Figure 2:**
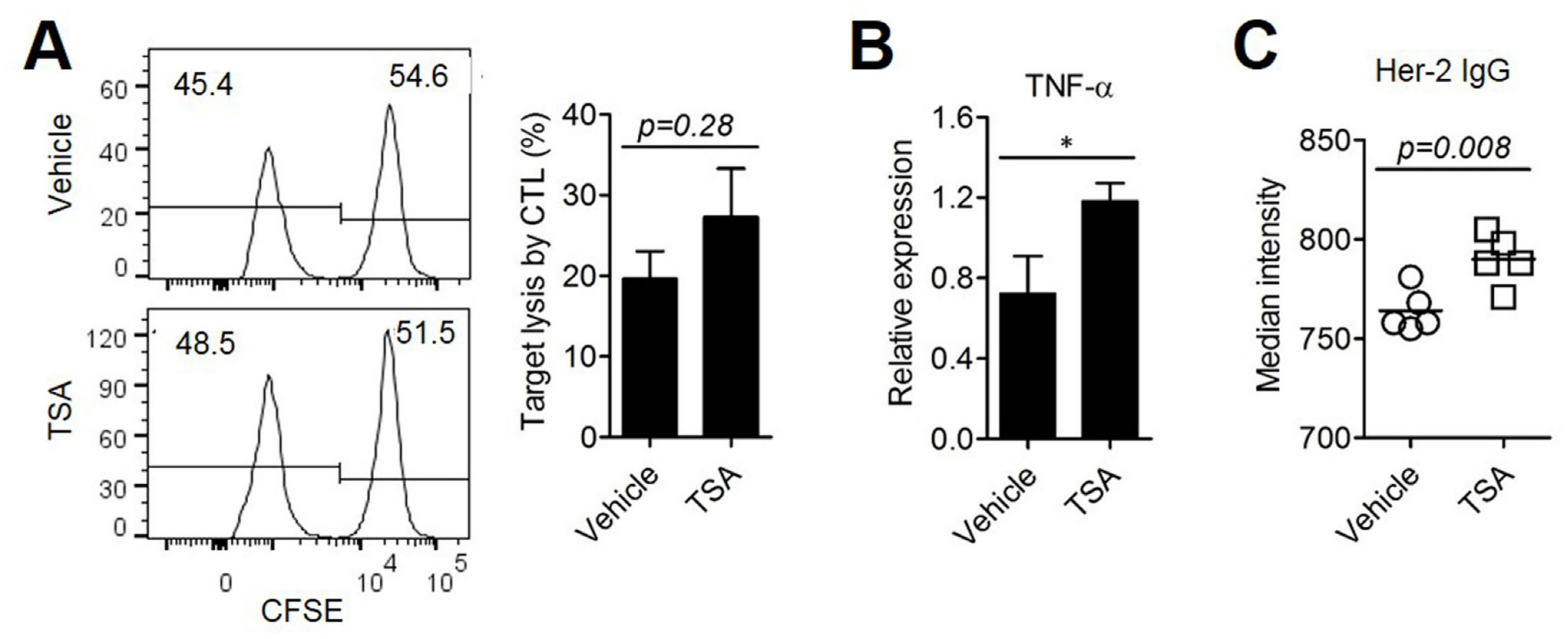
TSA treatment could enhance anti-tumor immunity **(A)** Specific lysis of hp63 (TYLPTNASL) peptide-loaded target cells was estimated by *in vivo* CTL (Student's *t*-*t*est; n=5 per group). **(B)** mRNA was isolated from the tumors and the mRNA levels of TNF-α were analyzed by RT-PCR (*p<0.05., Student's *t*-test; n=5 per group). **(C)** Her2-specific Abs were assessed from the sera of Her2/CT26 tumor-bearing mice treated with TSA or vehicle. Her2/CT26 cells were stained with each sera, followed by staining with FITC-conjugated secondary anti-IgG Abs (Student's *t*-test; n=5 per group).

To investigate whether TSA treatment enhanced anti-tumor immunity, we determined the level of Her2-specific CTL activity. BALB/c mice were subcutaneously (s.c.) injected with Her2/CT26 cells and Her2-specific lysis of hp63 (TYLPTNASL) peptide-pulsed target cells *in vivo* (Figure 2A). Although there were some CTLs against Her2, TSA treatment did not significantly alter the level of Her2-specific CTLs. However, the level of TNF-α mRNA, which may be critical for the induction of apoptosis in Her2/CT26 cells *in vivo*, was significantly increased in tumor tissues of TSA-treated mice compared to vehicle-treated mice (Figure 2B). We next assessed whether TSA administration in mice could enhance the amount of tumor-antigen-specific Ab production. To test this, we obtained sera from tumor-bearing mice treated with either TSA or vehicle. Abs generated in tumor-bearing mice were diluted as indicated and the specific binding of anti-Her2 Abs on the surface of Her2/CT26 cells was analyzed using flow cytometry analysis. Sera from tumor-bearing mice treated with TSA had higher MFI (mean fluorescence intensity) than vehicle-treated mice at 1:250 serum dilution, suggesting that Her2-specific Ab production was increased by TSA treatment (Figure 2C). These results suggest that TSA could inhibit the growth of colorectal cancer by enhancing several factors responsible for anti-tumor immunity.

### TSA loosens tumor suppressive microenvironment by decreasing CD4^+^ Treg and myeloid-derived suppressor cells

**Figure 3:**
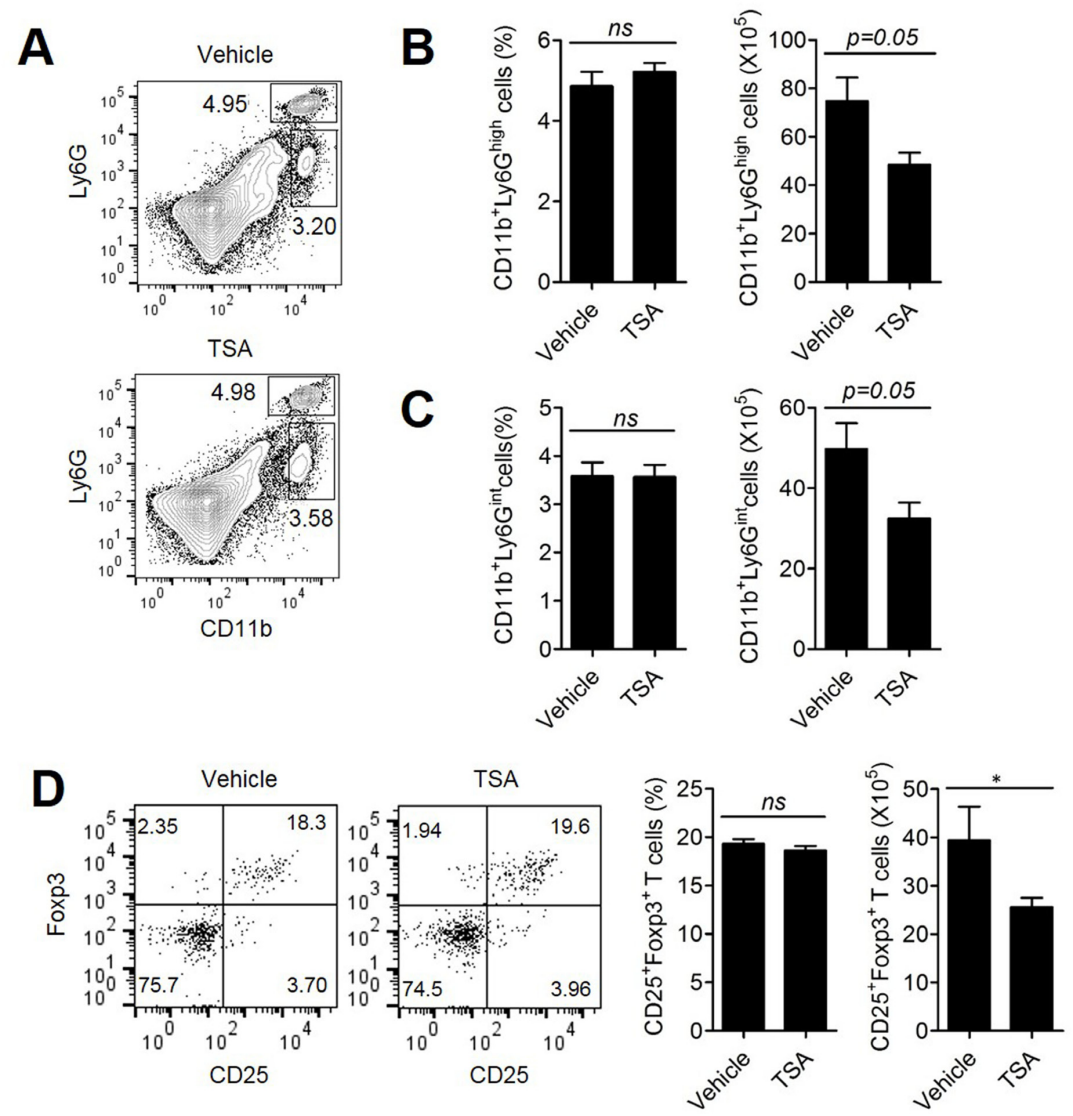
TSA treatment could attenuate the tumor-suppressive microenvironment Mice received s.c. injections of Her2/CT26 cells and were treated with TSA or vehicle. **(A)** Splenic myeloid cells obtained from the spleens of Her2/CT26 tumor-bearing mice were analyzed by flow cytometry. The percentage and number of CD11b^+^Ly6G^high^ **(B)** and CD11b^+^Ly6G^int^ cells **(C)** are shown (Student's *t*-test; n=5 per group). **(D)** For the determination of CD4^+^ Treg cells in the spleen of tumor-bearing mice, the Foxp3^+^CD25^+^ gated population among CD4^+^ T cells was analyzed (*p<0.01, ns; not significant, Student's *t*-test; n=5 per group).

Previously, we and others have shown that MDSCs, consisting of neutrophilic and monocytic cells, are present in the tumor microenvironment and are involved in tumor progression [25]. Therefore, we assessed the population of CD11b^+^Ly6G^+^ neutrophilic and CD11b^+^Ly6G^int^ monocytic MDSCs in the spleen of Her2/CT26 tumor-bearing mice after TSA or vehicle treatment (Figure 3A). Although there were no significant changes in the percentages of CD11b^+^Ly6G^+^ and CD11b^+^Ly6G^int^ cells between TSA- and Vehicle-treated groups, the number of CD11b^+^Ly6G^+^ and CD11b^+^Ly6G^int^ cells was reduced by TSA treatment compared to vehicle treatment (Figures 3A-3C).

We also assessed the level of regulatory T cells (Treg cells) that could inhibit the effector function of antitumor T cells. Although the percentage of CD25^+^FoxP3^+^ regulatory T cells among CD4^+^ T cells in the spleen of Her2/CT26 tumor-bearing mice was not changed significantly by the administration of TSA, TSA treatment significantly reduced the number of CD25^+^FoxP3^+^ Treg cells compared to vehicle treatment (Figure 3D). The decreased number of Treg cells and MDSCs in spleen of TSA-treated mice, compared to the vehicle-treated group could be because of the decreased number of total splenic CD4^+^ T cells associated with reduced tumor size (Supplementary Figure 1), rather than the direct inhibitory effects of TSA on Treg cells and MDSCs.

We next assessed the level of CD25^+^FoxP3^+^ Tregs and myeloid-derived suppressor cells in the tumor tissues. Mice were s.c. inoculated with 2×10^5^ Her2/CT26 tumor cells, and TSA was started to be given intraperitoneally (10mg/kg) for 3 times per every week as the average tumor volume reached 100 mm^3^ for 2 weeks. Total cells were isolated using methods which we previously reported [25]. CD45-expressing cells were gated for the analysis of MDSCs and Treg cells. TSA treatment significantly decreased the percentage and number of CD11b^+^Ly6C^+^ monocytic MDSCs in tumor tissue, whereas no significant alterations were not detected in those of CD11b^+^Ly6G^+^ neutrophilic MDSCs (Supplementary Figure 2A). In addition, we could find that the number of Treg cells expressing CD4, CD25, and FoxP3 were decreased in tumor tissue by TSA treatment (Supplementary Figure 2B).

Collectively, these results suggested that TSA could inhibit the growth of colorectal cancer by loosening the immune-suppressive microenvironment.

### TSA directly induces autophagy in tumor cells

**Figure 4:**
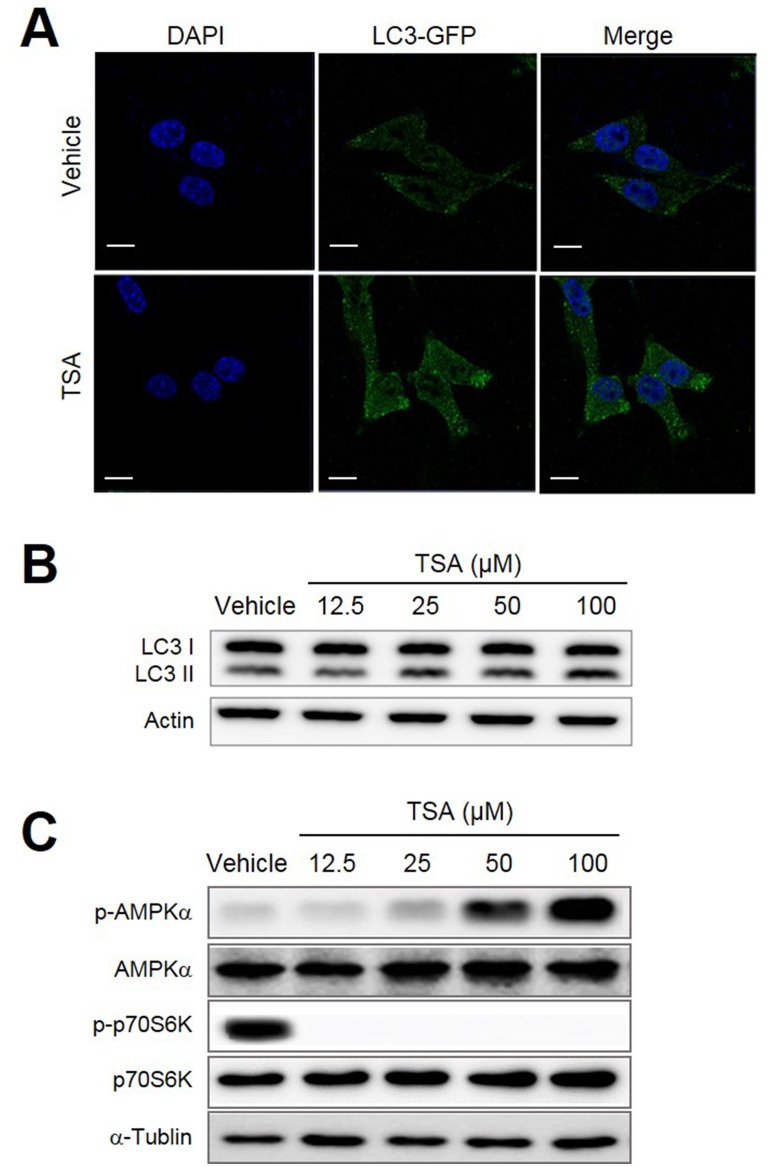
TSA induced autophagy within tumor cells **(A)** Her2/CT26 cells were treated with TSA and stained for LC3 using Abs, followed by addition of Alexa Fluor 488-conjugated goat anti-rabbit IgGs (shown in green). Cells were stained with DAPI to visualize the nuclei (shown in blue) and then analyzed for LC3 using confocal microscopy. **(B)** The conversion of LC3-I to LC3-II was assessed in TSA-treated Her2/CT26 cells using Western blots. **(C)** The protein levels of phosphorylated-AMPKα, AMPKα, phosphorylated p70S6K, p70S6K, and α-tubulin were assessed by western blot.

It was recently reported that some polyphenolic compounds induced autophagic cell death [26]. We, therefore, decided to test whether TSA could also induce autophagy in cancer cells. Her2/CT26 cells were treated with TSA for 24 h and cells were stained with Abs against LC3. With the use of confocal microscopy, we detected an increased level of LC3 puncta (green color) in cells treated with TSA compared to those treated with vehicle (Figure 4A). TSA treatment increased conversion of LC3-I to LC3-II in Her2/CT26 cells compared to cells treated with vehicle alone (Figure 4B).

The cumulative data suggest that polyphenolic compounds, including resveratrol, induce autophagy by regulating multiple signaling pathways involving AMPK and mTORC1. Therefore, we tested whether TSA induces autophagy through activation of AMPK or inhibition of mTORC1. As expected, TSA induced p-AMPK (T172) levels in a dose-dependent manner (Figure 4C). Remarkably, TSA completely inhibited mTORC1 activity as estimated by the p-p70S6K levels at the TSA concentrations tested (Figure 4C). These results suggest that TSA-mediated AMPK activation and inhibition of mTORC1 pathway might be associated with autophagy induction.

### TSA induced tumor cell death

**Figure 5:**
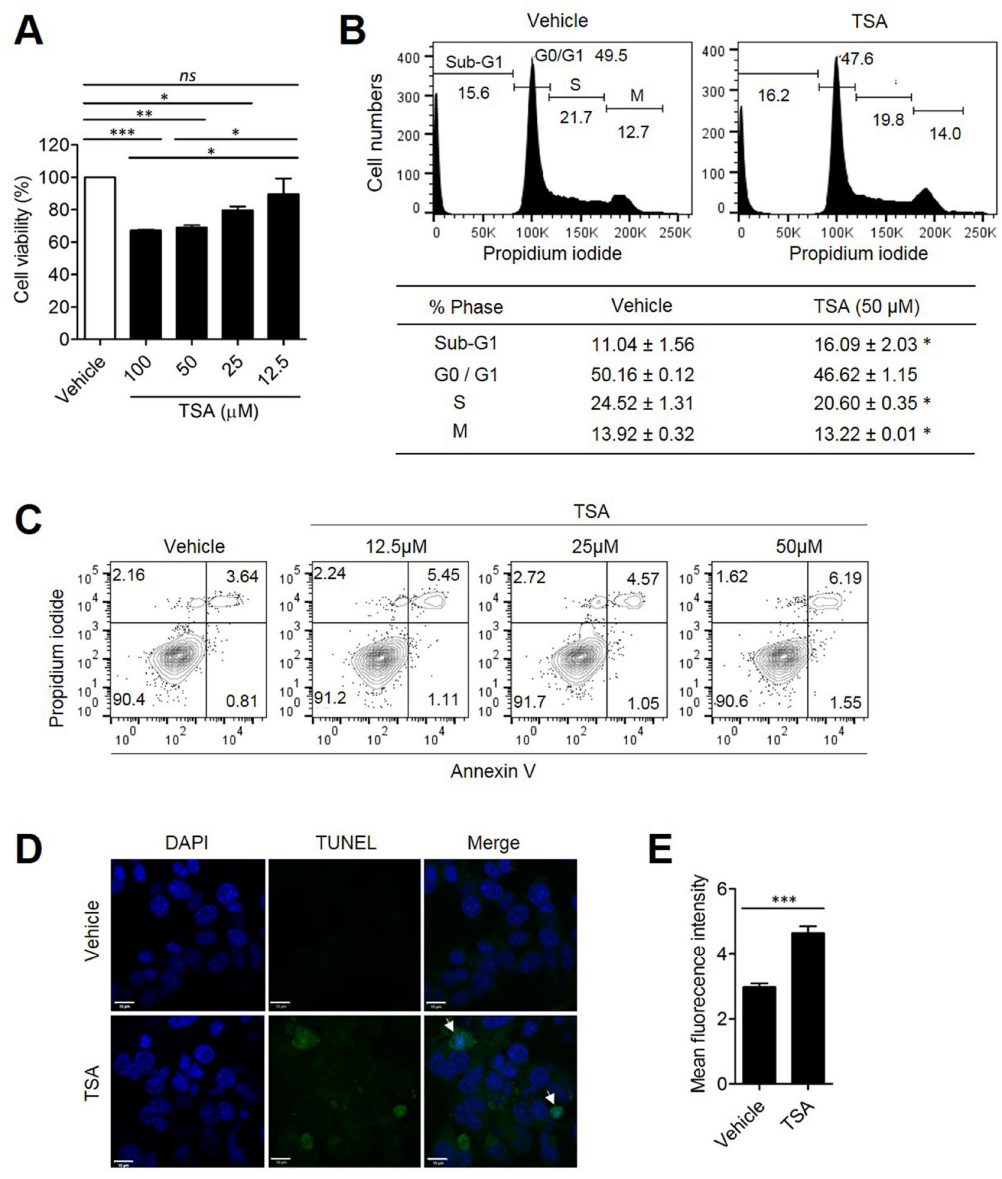
TSA directly induced tumor cell death Her2/CT26 cells were treated with the indicated concentration of TSA. **(A)** Cell viability was evaluated at 24 h after treatment and was determined based on the absorbance at 480 nm (*p<0.05, **p<0.01, and ***p<0.0001, ns; not significant, ANOVA). **(B)** Propidium iodide-labeled nuclei were analyzed for determination of cell cycle stage. The sub-G1 phase cells containing apoptotic populations were analyzed by flow cytometry (*p<0.01, two-tailed unpaired *t*-test). **(C)** Cells were incubated with 0, 12.5, 25, and 50 μM TSA for 24 h. Annexin-V- and propidium iodide-stained cells were analyzed for cell death using flow cytometry. **(D)** Her2/CT26 cells were treated with 100 μM TSA for 24 h and then stained using the TUNEL assay. Apoptotic cells were examined by confocal microscopy. **(E)** The mean green fluorescence intensity of TUNEL positive cells is summarized (***p<0.001, two-tailed unpaired *t*-test).

Because excessive autophagy can lead to cell death, we tested whether autophagy induction by TSA is associated with increased cell death. Her2/CT26 cells were incubated with TSA and cell viability was determined. The results show that TSA significantly decreased the viability of Her2/CT26 cell when treated with 12.5, 25, and 50 μM /ml of TSA for 24 h (Figure 5A). When the DNA content of tumor cells was monitored, TSA-treated cells had increased percentages of cells in the sub-G1 phase, in a dose-dependent manner, after 24 h of incubation (Figure 5B). The fragmentation of DNA, detected by the increased level of the sub-G1 phase of the cell cycle, could be an indicator of apoptotic cell death [27]. Furthermore, we found that TSA treatment induced a significant reduction of cells at the S-phase with cell cycle arrest at G1 phase (Figure 5B). To confirm whether TSA could induce cell death via the apoptosis pathway, Annexin V/PI staining was used to analyze Her2/CT26 cells treated with TSA for 24 h. Treatment of cells with TSA significantly increased the percentage of Annexin V/PI-double positive in comparison to cells treated with vehicle (Figure 5C), suggesting that TSA induced apoptotic cell death in Her2/CT26 cells. In addition, there was an increase in the number of TUNEL-positive cells when TSA treatment was administered compared to cells treated with vehicle alone, suggesting that TSA treatment increased apoptotic cell death (Figure 5D and 5E). We also checked the level of TUNEL-positive cells in tumor tissues of vehicle- or TSA-treated mice. Mice were s.c. inoculated with 2×10^5^ Her2/CT26 tumor cells, and TSA was started to be given intraperitoneally (10mg/kg) for 3 times per every week as the average tumor volume reached 100 mm^3^ for 2 weeks. As a result, we could find that the number of TUNEL-positive cells was highly increased by the TSA treatment (Supplementary Figure 3), suggesting that apoptotic cell death was increased by TSA treatment.

Collectively, the results indicate that TSA could directly inhibit tumor growth by causing autophagy activation and apoptotic cell death. In turn, the immunogenic death of tumor cells could reduce the number of tumor suppressive immune cells and subsequently favor the induction of anti-tumor immunity.

### Combined treatment of TSA with docetaxel shows additive anti-cancer effect

**Figure 6:**
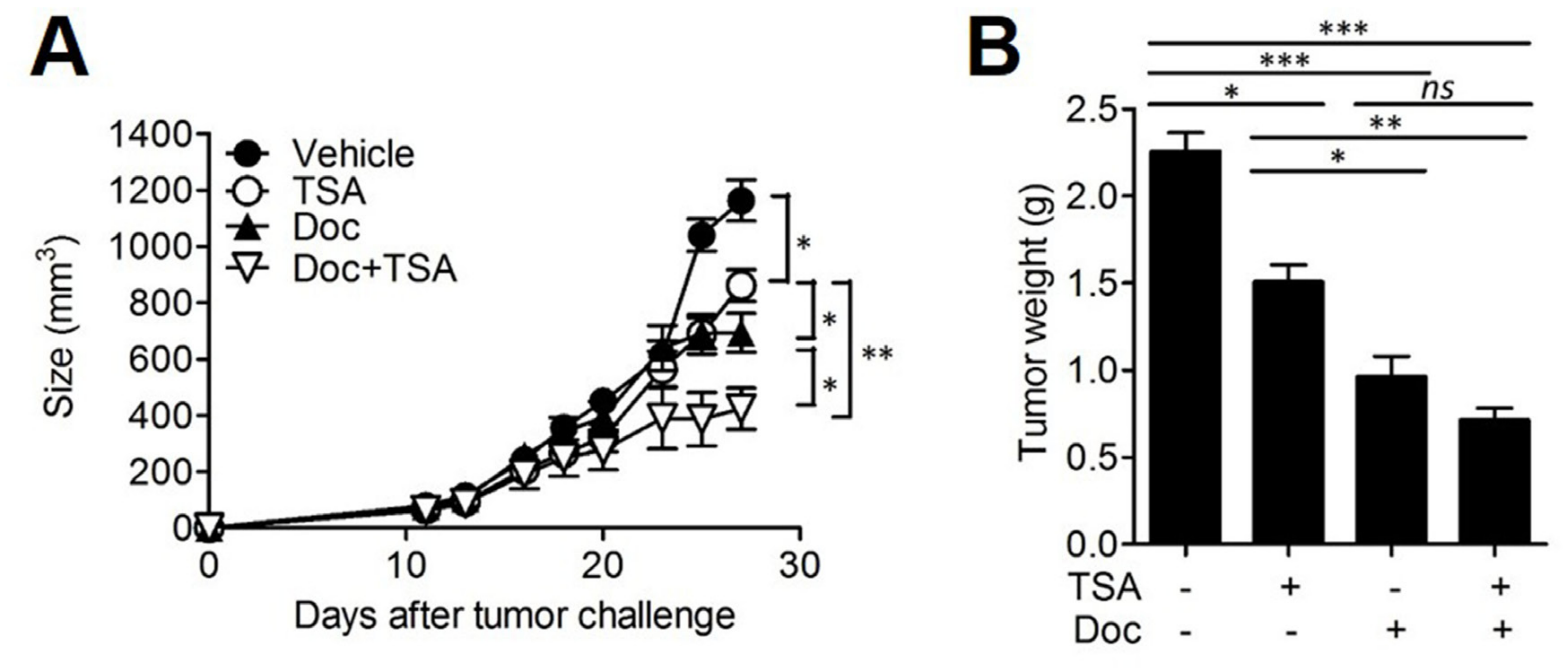
Combined therapy with TSA and docetaxel enhanced anti-cancer activity **(A)** BALB/c mice received *s.c*. injections of 2×10^5^ Her2/CT26 tumor cells. Mice were treated with each or both of TSA and docetaxcel as the tumor size reached 50~100 mm^3^. The growth of the tumor was monitored (*p<0.05, **p<0.01, ANOVA; n=5 per group, ANOVA). **(B)** The weight of the tumor was measured at 4 weeks after tumor challenge (*p<0.05, **p<0.01, and ***p<0.001; ANOVA; n=5 per group).

Docetaxel is a well-characterized anti-neoplastic drug currently used for the treatment of several solid tumors including colon cancer and breast cancer [28]. We assessed whether the combined treatment of TSA with docetaxel could increase the anti-cancer effect of docetaxel. When the tumor size reached 50-100 mm^3^, BALB/c mice were s.c. injected with 2×10^5^ Her2/CT26 cells and treated with (1) TSA, (2) docetaxel, or, (3) TSA combined with docetaxel. We found that the intravenously (i.v.) injection of docetaxel alone could significantly inhibit tumor growth compared to vehicle-treated mice (Figure 6A). We also confirmed that TSA treatment alone also showed significant antitumor effect. Furthermore, we showed that the combined treatment of mice with docetaxel and TSA successfully inhibited tumor growth to a greater extent than docetaxel alone (Figure 6A), and this finding was supported by the reduced tumor weight at the end of the treatment (Figure 6B). Taken together, these data demonstrate that TSA exhibits direct anti-cancer effects and that these effects can be further strengthened by co-administration of docetaxel.

## DISCUSSION

Scirpusin A is a hydroxystilbene dimer and was first isolated from the rhizoma of *Scirpus fluviatilis* (TORR.) A. GRAY (Cyperaceae) together with scirpusin B, resveratrol, and 3, 3’, 4, 5’-tetrahydroxystilbene [23]. It has therapeutic potential for the treatment of Alzheimer's disease because of its inhibitory effects on amyloid-beta-peptide aggregation and its beta-secretase inhibitory activity [22]. Scirpusin A also possesses the ability to quench singlet oxygen and DNA protective activity [23]. Scirpusin B, on the other hand, is a dimer of piceatannol (3,3',4,5'-tetrahydroxy-trans-stilbene), a hydroxylated analog of resveratrol, and known to have vasorelaxing activity [24]. As shown in the Supplementary Figure 4, trans-scirpusin B has one more hydroxyl group in the phenyl ring as compared with the structure of TSA. Hence, this structural difference seems to make difference in bioactivity. Recent studies suggest that both scirpusin A and scirpusin B have anti-adipogenic and anti-obesity potential. However, the anti-neoplastic activity of scirpusin A has not been reported.

In the current study, we used TSA isolated from *Borassus flabellifer L*.. Because TSA is a polymeric form of resveratrol, we assumed that TSA shares some biological activity with resveratrol, and specifically anti-cancer activity. It was also known that resveratrol showed anti-tumor effect via inhibition of carcinogenic activation, induction of apoptosis, and attenuation of pro-inflammatory signaling associated with cancer progression [29]. On the contrary, the current study is the first report suggesting the anti-tumor effect of TSA. Our result suggests that TSA induced autophagy activation and apoptotic cell death. We presume that activation of AMPK might associate with the induction of apoptosis as well as autophagy activation. Interestingly, a recent paper showed that resveratrol could increase the sensitivity of pancreatic cancer cells to gemcitabine by activating AMPK [30]. In our study, likewise, we also found that the combined treatment of TSA with docetaxel increased their synergistic anti-cancer activity in colon cancer cells *in vivo*. Because AMPK activation is also associated with autophagy activation, we could not determine whether the antitumor effect was dependent on AMPK activation or autophagy-induced apoptosis. Autophagy-induced cell death is a cell-intrinsic mechanism to remove abnormal cells under excessive stress. Rapidly proliferating tumor cells under hypoxic conditions undergo autophagy activation to survive. However, unabated activation of autophagy sometimes results in apoptotic cell death. Thus, by inducing unrestrained autophagy in tumor cells, we can expect anti-cancer activity. In this regard, TSA could elicit apoptosis in cancer cells, similar to resveratrol, by inducing autophagy. Although we showed that TSA induced autophagy activation and apoptosis in colon cancer cells, there could be other mechanisms than autophagic cell death to induce cancer cell death, which include necrosis and necroptosis.

To address whether docetaxcel (Doc) could induce autophagy activation as TSA did, we also confirmed the levels of LC3s in Her2/CT26 cells. The level of LC3-II was increased by TSA, but not by docetaxcel (Supplementary Figure 5A), suggesting that docetaxcel might not induced autophagy activation in the concentration we tested. We also assessed the cell viability after treating cells with TSA with or without chloroquine (CQ), which inhibits autophagy by inhibiting fusion of autophagosome with lysosome. We found that treatment of colon cancer cells with CQ inhibited cell death triggered by TSA (Supplementary Figure 5B).

It is difficult to completely remove all residual cancer cells by chemotherapy. In the current study, we propose the use of TSA as a supplementary treatment to enhance the anticancer effect of docetaxel by inducing cancer cell death via the autophagy activation and apoptosis induction.

## MATERIALS AND METHODS

### Cell line and mice

Murine CT26 colon cancer cells expressing human Her-2/neu (Her2/CT26) were used for *in vitro* and *in vivo* studies [25]. Her2/CT26 was maintained in Dulbecco's Modified Eagle's Medium (DMEM) supplemented with 10% fetal bovine serum and 1% antibiotic-antimycotic solution. Wild-type inbred BALB/c mice were purchased from KOATECH Bio (Pyeongtaek, Korea). Mice used were between the ages of 6 and 7 weeks. Mice were maintained under specific pathogen-free conditions within the experimental facility at the animal center of Kangwon National University (KW-140811-2). To establish tumors, 2×10^5^ Her2/CT26 tumor cells were injected *s.c*. into the left flank of BALB/c. Tumor growth was measured with calipers 3 times per week. Ten mg/kg TSA was administered via i.p. injection 3 times per week and 20 mg/kg docetaxel was given intravenously (once a week until the tumor volume reached 50-100 mm^3^).

### Preparation of TSA

TSA was isolated from the roots of *Borassus flabellifer* L.collected in Cambodia. Briefly, the dried roots of *B. gomutus* (1.4 kg) were extracted with 100% MeOH and evaporated in vacuo, yielding 81.7 g of crude extract. The crude extract was partitioned with EtOAc, BuOH, and water, and the different fractions were collected. The EtOAc-soluble extract (25.6 g) was separated using chromatography on a silica gel column with a gradient mixture of chloroform and methanol (100:1 to 1:1) and produced 18 sub-fractions (AP01-AP18). Among them, fraction 10 was further fractionated using medium pressured liquid chromatography (MPLC) and a gradient solvent of methanol and water (25:75 to 100:0) into 10 subfractions (AP1001-AP1010). In AP1010, crude precipitates were obtained and then recrystallized (MeOH) to yield TSA. The structure of TSA was confirmed by comparing NMR and MS data of the precipitate with published data [31].

### Cell viability assay

Cell viability was measured using the CCK-8 (cell counting kit-8, Dojindo, Gaithersburg, MD) as previously described [32]. Her2/CT26 cells were seeded at a concentration of 2×10^5^ cells/well in 96-well plates and incubated for 24 h at 37°C with 5% CO_2_. The next day, cells were treated with indicated concentration of TSA. After incubation for 6 h and 24 h, Her2/CT26 cells were washed with PBS, 10 μl CCK-8 solution were added to each well and plates were incubated for another 4 h at 37°C with 5% CO_2_. Cell viability was determined by reading absorbance at 450 nm using a SpectraMax I3 microplate reader (Molecular Devices, Palo Alto, CA) with a reference absorbance at 620 nm to correct for nonspecific background values.

### *In vivo* cytotoxic T lymphocyte assay

The *in vivo* cytotoxicity was assessed as described elsewhere [25]. Briefly, syngeneic splenocytes were divided into equal numbers and either loaded with 1 μg/mL of cytotoxic T lymphocyte (CTL) epitope peptide (human HER2/neu p63 [TYLPTNASL]) or left unpulsed. Peptide-pulsed cells (CFSE^high^) were labeled with 20 μM chloromethyl fluorescein diacetate succinimidyl ester (CFSE; Invitrogen, Carlsbad, CA) and unpulsed cells were labeled with 2 μM CFSE (CFSE^low^). Equal numbers of CFSE^high^ and CFSE^low^ cells were mixed and injected intravenously (i.v.) into groups of mice. After 24 h, the splenocytes from treated mice were analyzed using flow cytometry to assess antigenic peptide-specific target lysis. The specific lysis was calculated as follows: r (ratio) = (% CFSE ^high^/CFSE ^low^) and % lysis= [1-(r _unpulsed_/r _pulsed_)]x100.

### Quantitative real-time PCR

For real-time PCR analysis, total RNA was isolated from tumor tissue using the RNA Extraction Mini kit (Qiagen, Germany) and reverse transcription was performed using the cDNA Synthesis Mini Kit (iNtRON, Korea) as previously described [33]. The quantitative real-time PCR was carried out using THUNDERBIRD™ SYBR QPCR Mix (Toyobo, Japan). Primers used were: TNF-α Forward Primer: 5’-TGG GAG TAG ACA AGG TAC AAC CC-3’, TNF-α Reverse Primer: 5’-CAT CTT CTC AAA ATT CGA GTG ACA A-3’, β-actin Forward Primer: 5’-CCT AGG CAC CAG GGT GTG AT-3’ and β-actin Reverse Primer: 5’-TCT CCA TGT CGT CCC AGT TG-3’. The cycling conditions were as follows: 95°C for 3 min, followed by 39 cycles of 95°C for 30 s, 55°C for 30 s, and 72°C for 30 s.

### Flow cytometry

Cells were collected from the spleen of mice and stained with the following Abs: fluorescein isothiocyanate (FITC)-conjugated anti-CD11b, and, phycoerythrin-Cy7 tandem-conjugated anti-Ly6G. After staining, cells were analyzed for their expression of CD11b and Ly6G. Regulatory T cells were stained with the following Abs: anti-CD4-FITC, anti-CD25-PE, anti-FoxP3-APC All Abs used for flow cytometry analysis were purchased from BD Biosciences (San Jose, CA). Ab response analysis was performed as described previously [34]. To evaluate Her-2/neu-specific Ab production, the amount of murine Ab binding to Her2/CT26 cells was determined using flow cytometry. Briefly, Her2/CT26 cells were stained with diluted sera containing anti-Her2 polyclonal Abs, and then FITC-labeled goat anti-mouse IgG Abs (BD Bioscience, San Diego, CA) were added to detect cell-bound murine IgG. The number of cells undergoing apoptosis was determined using an Annexin V/Propidium iodide (PI) apoptosis kit (BD Biosciences, San Diego, CA). For Annexin V/PI staining, cells were harvested, washed twice with cold PBS and then resuspended in 1X binding buffer at a concentration of 1×10^6^ cell/ml. Annexin V-FITC conjugate and PI solution were added to the cells, gently vortexed, and incubated for 15 min at RT (room temperature) in the dark. Cells were read using FACSVerse (BD Bioscience, San Diego, CA) and the data were analyzed using BD FACSuite software application.

### Staining for apoptosis using TUNEL assay and confocal microscopy

Apoptotic cell death was determined using the TUNEL (terminal deoxynucleotidyl transferase dUTP nick end labeling) assay kit (Chemicon, Billerica, MA) following the manufacturer's instructions. For autophagy analysis, cells were fixed with 4% paraformaldehyde for 10 min at RT (room temperature). Cells were stained with anti-LC3 Abs (Cell Signaling Technology, Danvers, MA) and incubated overnight at 4°C. After washing with PBS 3X, anti-rabbit IgG Abs conjugated with Alexa Fluor 488 (Abcam, Cambridge, MA) were added and incubated for 2 h at RT. Nuclei were stained with DAPI (4',6-diamidino-2-phenylindole) and cells were analyzed using confocal microscopy (SM880 with Airyscan, Zeiss, NY).

### Western blot analysis

Cells were appropriately treated, and total cell lysates and nuclear extracts were prepared as previously described [35]. Total protein lysates from cells were prepared by sonicating cells in lysis buffer (iNtRON, Korea). Equal amounts of lysates were boiled at 100°C and resolved by 10-12% sodium dodecyl sulfate-polyacrylamide gel electrophoresis (SDS-PAGE). Proteins were transferred to polyvinylidene difluoride (PVDF) membranes (Millipore, Billerica, MA) and then blocked with 5% milk in Tris-buffered saline and 0.1% Tween 20. The membranes were incubated overnight with primary Abs against LC3, AMPKα, phosphorylated AMPKα, p70S6K, phosphorylated p70S6K, and β-actin (all from Cell Signaling Technology, Danvers, MA), and proteins were detected with goat-anti-rabbit Abs conjugated with horseradish peroxidase (HRP) (Cell Signaling Technology, Danvers, MA). Proteins were detected using the enhanced chemiluminescence (ECL) method with femtoLUCENT ™ PLUS-HRP (G-biosciences, St. Louis, MO).

### Statistical analysis

GraphPad prism software (GraphPad, La Jolla, CA) was used for statistical analysis. Student's *t*-tests were used to compare mean values between 2 groups, while a one-way analysis of variance with Tukey's HSD post-hoc test was used for comparisons among more than 2 groups. Values of P < 0.05 were considered significant.

## SUPPLEMENTARY MATERIALS FIGURES



## Abbreviations

TSA: trans-Scirpusin A
CTLs: cytotoxic T lymphocytes
MDSCs: myeloid-derived suppressor cells
Treg cells: regulatory T cells
AMPK: AMP-activation protein kinase

## References

[1] Brglez Mojzer E, Knez Hrncic M, Skerget M, Knez Z, Bren U (2016). Polyphenols: extraction methods, antioxidative action, bioavailability and anticarcinogenic effects. Molecules.

[2] Smeriglio A, Galati EM, Monforte MT, Lanuzza F, D'Angelo V, Circosta C (2016). Polyphenolic compounds and antioxidant activity of cold-pressed seed oil from Finola Cultivar of Cannabis sativa L. Phytother Res.

[3] Feng Y, Yu YH, Wang ST, Ren J, Camer D, Hua YZ, Zhang Q, Huang J, Xue DL, Zhang XF, Huang XF, Liu Y (2016). Chlorogenic acid protects D-galactose-induced liver and kidney injury via antioxidation and anti-inflammation effects in mice. Pharm Biol.

[4] Machova Urdzikova L, Karova K, Ruzicka J, Kloudova A, Shannon C, Dubisova J, Murali R, Kubinova S, Sykova E, Jhanwar-Uniyal M, Jendelova P (2016). The anti-inflammatory compound curcumin enhances locomotor and sensory recovery after spinal cord injury in rats by immunomodulation. Int J Mol Sci.

[5] Riccioni G, Gammone MA, Tettamanti G, Bergante S, Pluchinotta FR, D'Orazio N (2015). Resveratrol and anti-atherogenic effects. Int J Food Sci Nutr.

[6] Abdal Dayem A, Choi HY, Yang GM, Kim K, Saha SK, Cho SG (2016). The anti-cancer effect of polyphenols against breast cancer and cancer stem cells: molecular mechanisms. Nutrients.

[7] Mocanu MM, Nagy P, Szollosi J (2015). Chemoprevention of breast cancer by dietary polyphenols. Molecules.

[8] Fantini M, Benvenuto M, Masuelli L, Frajese GV, Tresoldi I, Modesti A, Bei R (2015). *In vitro* and *in vivo* antitumoral effects of combinations of polyphenols, or polyphenols and anticancer drugs: perspectives on cancer treatment. Int J Mol Sci.

[9] Weiskirchen S, Weiskirchen R (2016). Resveratrol: How much wine do you have to drink to stay healthy?. Adv Nutr.

[10] Weber K, Schulz B, Ruhnke M (2011). Resveratrol and its antifungal activity against Candida species. Mycoses.

[11] Hwang D, Lim YH (2015). Resveratrol antibacterial activity against Escherichia coli is mediated by Z-ring formation inhibition via suppression of FtsZ expression. Sci Rep.

[12] Tome-Carneiro J, Gonzalvez M, Larrosa M, Yanez-Gascon MJ, Garcia-Almagro FJ, Ruiz-Ros JA, Tomas-Barberan FA, Garcia-Conesa MT, Espin JC (2013). Resveratrol in primary and secondary prevention of cardiovascular disease: a dietary and clinical perspective. Ann N Y Acad Sci.

[13] Hausenblas HA, Schoulda JA, Smoliga JM (2015). Resveratrol treatment as an adjunct to pharmacological management in type 2 diabetes mellitus--systematic review and meta-analysis. Mol Nutr Food Res.

[14] Baek SH, Ko JH, Lee H, Jung J, Kong M, Lee JW, Lee J, Chinnathambi A, Zayed ME, Alharbi SA, Lee SG, Shim BS, Sethi G (2016). Resveratrol inhibits STAT3 signaling pathway through the induction of SOCS-1: role in apoptosis induction and radiosensitization in head and neck tumor cells. Phytomedicine.

[15] Saud SM, Li W, Morris NL, Matter MS, Colburn NH, Kim YS, Young MR (2014). Resveratrol prevents tumorigenesis in mouse model of Kras activated sporadic colorectal cancer by suppressing oncogenic Kras expression. Carcinogenesis.

[16] Shrotriya S, Tyagi A, Deep G, Orlicky DJ, Wisell J, Wang XJ, Sclafani RA, Agarwal R, Agarwal C (2015). Grape seed extract and resveratrol prevent 4-nitroquinoline 1-oxide induced oral tumorigenesis in mice by modulating AMPK activation and associated biological responses. Mol Carcinog.

[17] Bognar E, Sarszegi Z, Szabo A, Debreceni B, Kalman N, Tucsek Z, Sumegi B, Gallyas F (2013). Antioxidant and anti-inflammatory effects in RAW264.7 macrophages of malvidin, a major red wine polyphenol. PLoS One.

[18] Berrougui H, Grenier G, Loued S, Drouin G, Khalil A (2009). A new insight into resveratrol as an atheroprotective compound: inhibition of lipid peroxidation and enhancement of cholesterol efflux. Atherosclerosis.

[19] Leonard SS, Xia C, Jiang BH, Stinefelt B, Klandorf H, Harris GK, Shi X (2003). Resveratrol scavenges reactive oxygen species and effects radical-induced cellular responses. Biochem Biophys Res Commun.

[20] Aggarwal BB, Bhardwaj A, Aggarwal RS, Seeram NP, Shishodia S, Takada Y (2004). Role of resveratrol in prevention and therapy of cancer: preclinical and clinical studies. Anticancer Res.

[21] Meng J, Guo F, Xu H, Liang W, Wang C, Yang XD (2016). Combination therapy using co-encapsulated resveratrol and paclitaxel in liposomes for drug resistance reversal in breast cancer cells *in vivo*. Sci Rep.

[22] Riviere C, Papastamoulis Y, Fortin PY, Delchier N, Andriamanarivo S, Waffo-Teguo P, Kapche GD, Amira-Guebalia H, Delaunay JC, Merillon JM, Richard T, Monti JP (2010). New stilbene dimers against amyloid fibril formation. Bioorg Med Chem Lett.

[23] Kong Q, Ren X, Jiang L, Pan Y, Sun C (2010). Scirpusin A, a hydroxystilbene dimer from Xinjiang wine grape, acts as an effective singlet oxygen quencher and DNA damage protector. J Sci Food Agric.

[24] Sano S, Sugiyama K, Ito T, Katano Y, Ishihata A (2011). Identification of the strong vasorelaxing substance scirpusin B, a dimer of piceatannol, from passion fruit (Passiflora edulis) seeds. J Agric Food Chem.

[25] Lee BR, Chang SY, Hong EH, Kwon BE, Kim HM, Kim YJ, Lee J, Cho HJ, Cheon JH, Ko HJ (2014). Elevated endoplasmic reticulum stress reinforced immunosuppression in the tumor microenvironment via myeloid-derived suppressor cells. Oncotarget.

[26] Hasima N, Ozpolat B (2014). Regulation of autophagy by polyphenolic compounds as a potential therapeutic strategy for cancer. Cell Death Dis.

[27] Rinner B, Li ZX, Haas H, Siegl V, Sturm S, Stuppner H, Pfragner R (2009). Antiproliferative and pro-apoptotic effects of Uncaria tomentosa in human medullary thyroid carcinoma cells. Anticancer Res.

[28] Song SY, Kim KP, Jeong SY, Park J, Park J, Jung J, Chung HK, Lee SW, Seo MH, Lee JS, Jung KH, Choi EK (2016). Polymeric nanoparticle-docetaxel for the treatment of advanced solid tumors: phase I clinical trial and preclinical data from an orthotopic pancreatic cancer model. Oncotarget.

[29] Whitlock NC, Baek SJ (2012). The anticancer effects of resveratrol: modulation of transcription factors. Nutr Cancer.

[30] Jiang Z, Chen X, Chen K, Sun L, Gao L, Zhou C, Lei M, Duan W, Wang Z, Ma Q, Ma J (2016). YAP inhibition by resveratrol via activation of AMPK enhances the sensitivity of pancreatic cancer cells to gemcitabine. Nutrients.

[31] Morikawa T, Xu F, Matsuda H, Yoshikawa M (2010). Structures of novel norstilbene dimer, longusone A, and three new stilbene dimers, longusols A, B, and C, with antiallergic and radical scavenging activities from Egyptian natural medicine Cyperus longus. Chem Pharm Bull (Tokyo).

[32] Song JH, Kwon BE, Jang H, Kang H, Cho S, Park K, Ko HJ, Kim H (2015). Antiviral activity of chrysin derivatives against coxsackievirus B3 in vitro and in vivo. Biomol Ther (Seoul).

[33] Kim YI, Yang JY, Ko HJ, Kweon MN, Chang SY (2014). Shigella flexneri inhibits intestinal inflammation by modulation of host sphingosine-1-phosphate in mice. Immune Netw.

[34] Kim YJ, Ko HJ, Kim YS, Kim DH, Kang S, Kim JM, Chung Y, Kang CY (2008). alpha-Galactosylceramide-loaded, antigen-expressing B cells prime a wide spectrum of antitumor immunity. Int J Cancer.

[35] Kim HJ, Kim J, Kang KS, Lee KT, Yang HO (2014). Neuroprotective effect of chebulagic acid via autophagy induction in SH-SY5Y cells. Biomol Ther (Seoul).

