# Supplementary material for: Trans-scirpusin A showed antitumor effects via autophagy activation and apoptosis induction of colorectal cancer cells
Source: Oncotarget. 2017 Apr 24;8(25):41401–11. doi: 10.18632/oncotarget.17388 (PMC5522333; doi:10.18632/oncotarget.17388)
Supplement: Supplementary file 1 [file oncotarget-08-41401-s001.pdf]

# *Trans*-scirpusin A showed antitumor effects via autophagy activation and apoptosis induction of colorectal cancer cells

## SUPPLEMENTARY MATERIALS

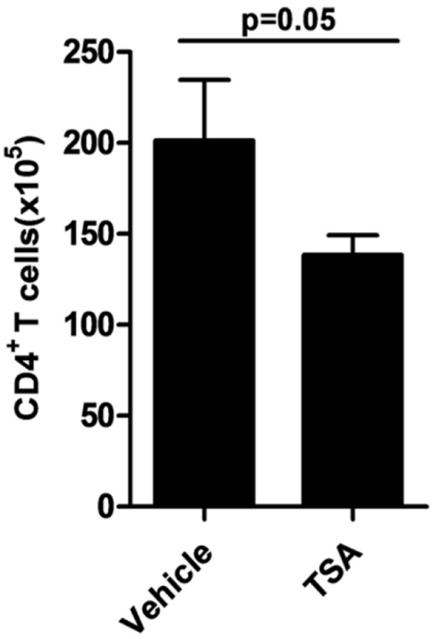


**Supplementary Figure 1: Number of CD4$^+$ T cell in spleen treated with TSA.** Mice received s.c. injections of Her2/CT26 cells and were treated with TSA or vehicle. CD4$^+$ T cells obtained from the spleens of Her2/CT26 tumor-bearing mice were analyzed by flow cytometry.

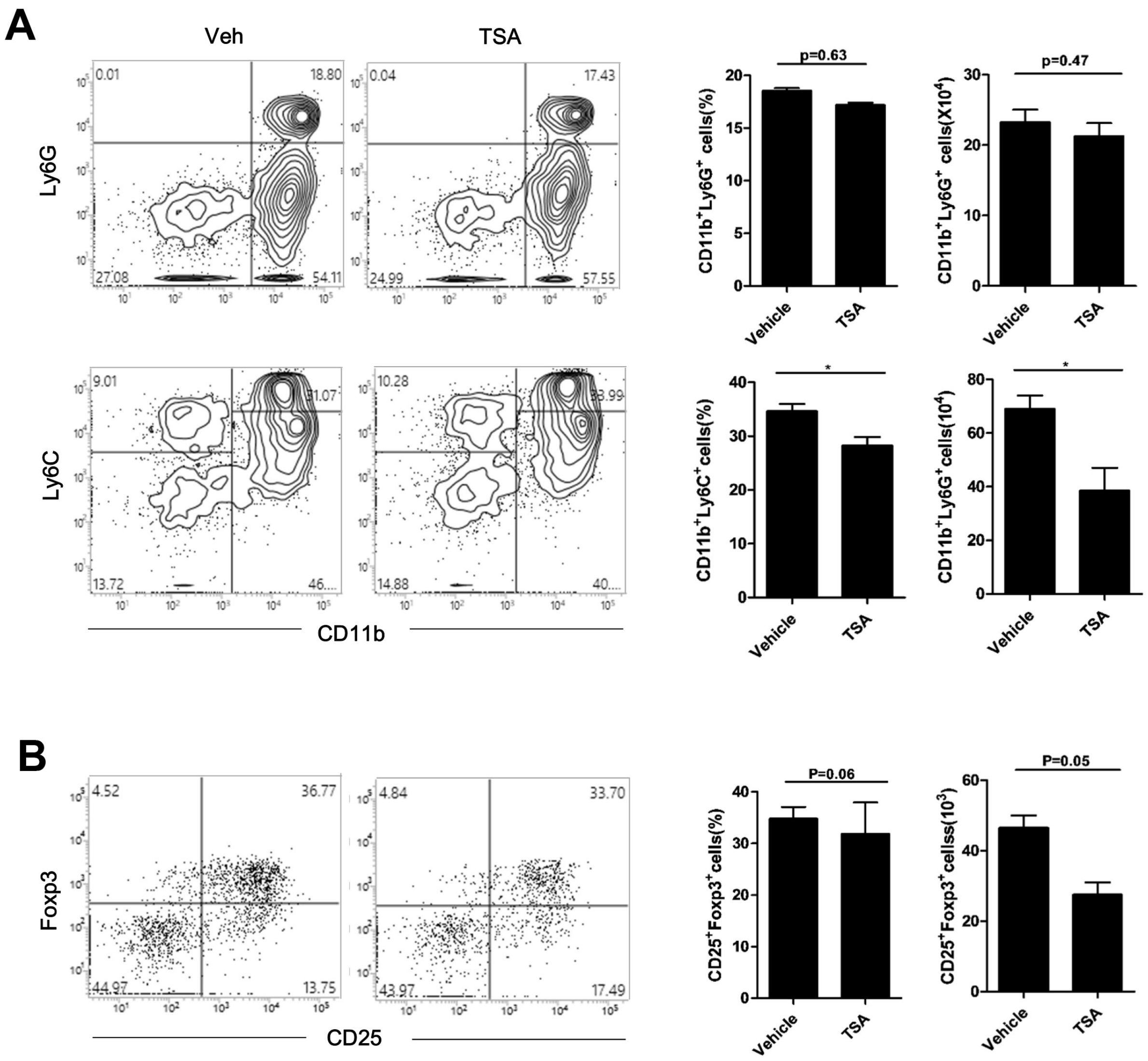


**Supplementary Figure 2: TSA treatment could attenuate the immune-suppressive microenvironment in tumor tissue.** Mice received s.c. injections of Her2/CT26 cells and were treated with TSA or vehicle. The MDSCs and Treg cells in tumor tissue were analyzed by flow cytometry after being gated by CD45 expression. **(A)** The percentage and number of CD11b$^{+}$Ly6G$^{+}$ and CD11b$^{+}$Ly6C$^{+}$ cells are shown (Two-tailed unpaired *t*-test). **(B)** For the determination of CD4$^{+}$ Treg cells in the tumor tissue, the Foxp3$^{+}$CD25$^{+}$ gated population among CD4$^{+}$ T cells was analyzed (Two-tailed unpaired *t*-test).

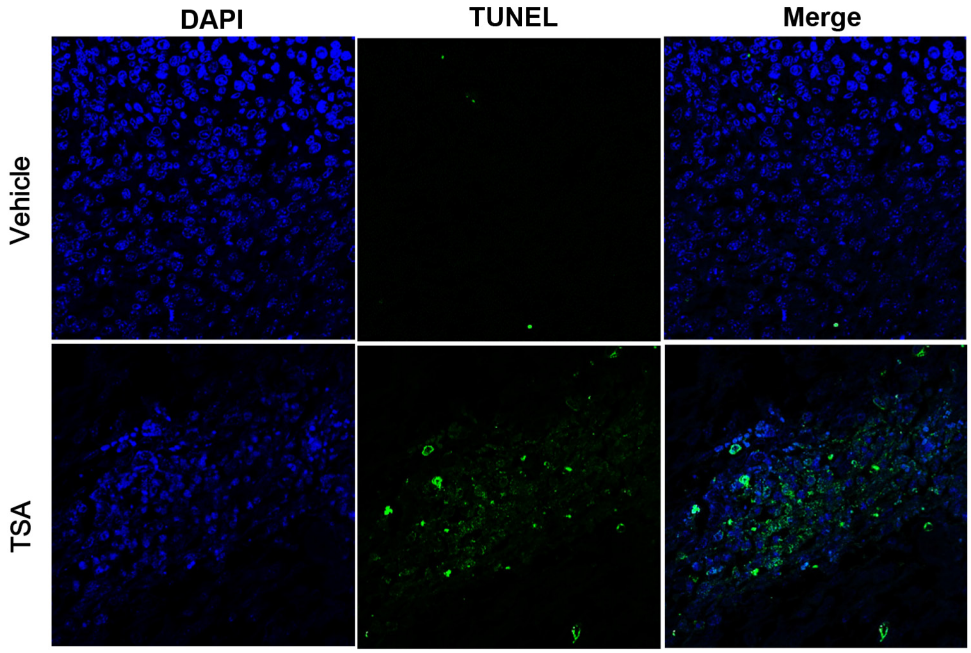


**Supplementary Figure 3: TSA-induced apoptotic cell death in tumor tissue.** BALB/c mice received *s.c.* injections of 2×$10^5$ Her2/CT26 tumor cells and TSA was started to be given by intraperitoneally (10mg/kg) for 3 times per every week as the average tumor volume reached 100 $mm^3$ for 2 weeks. The isolated tumor tissue and stained it using TUNEL assay. Apoptotic cells were examined by confocal microscopy.

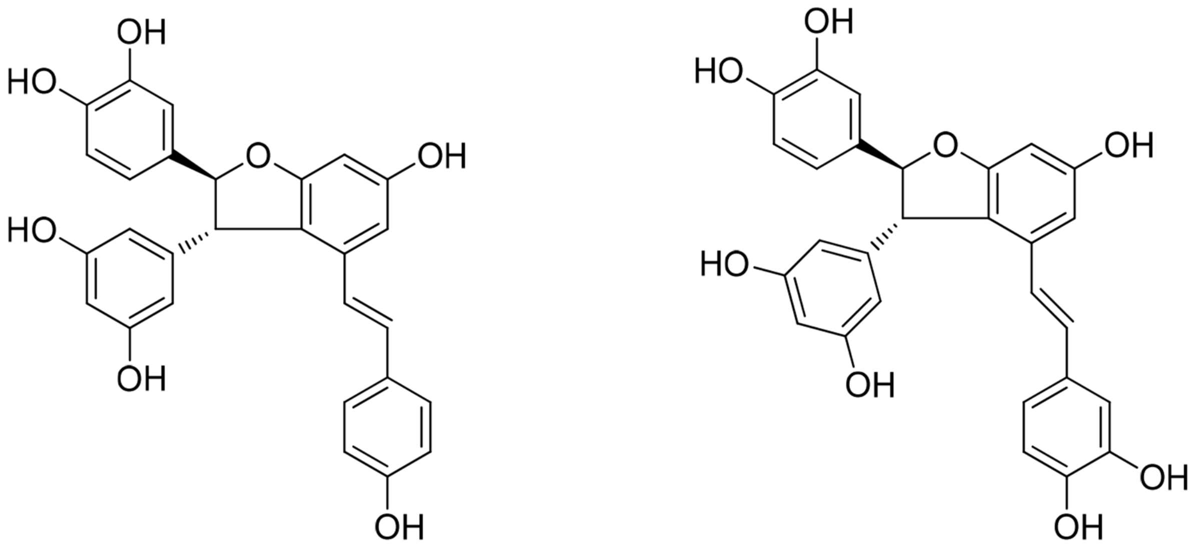

trans-scirpusin A trans-scirpusin B

**Supplementary Figure 4: The Structure of trans-scirpusin A (TSA) and trans-scirpusin B.** trans-scirpusin B has one more hydroxyl group in the phenyl ring as compared with the structure of trans-scirpusin A. Hence, this structural difference seems to make difference in bioactivity.

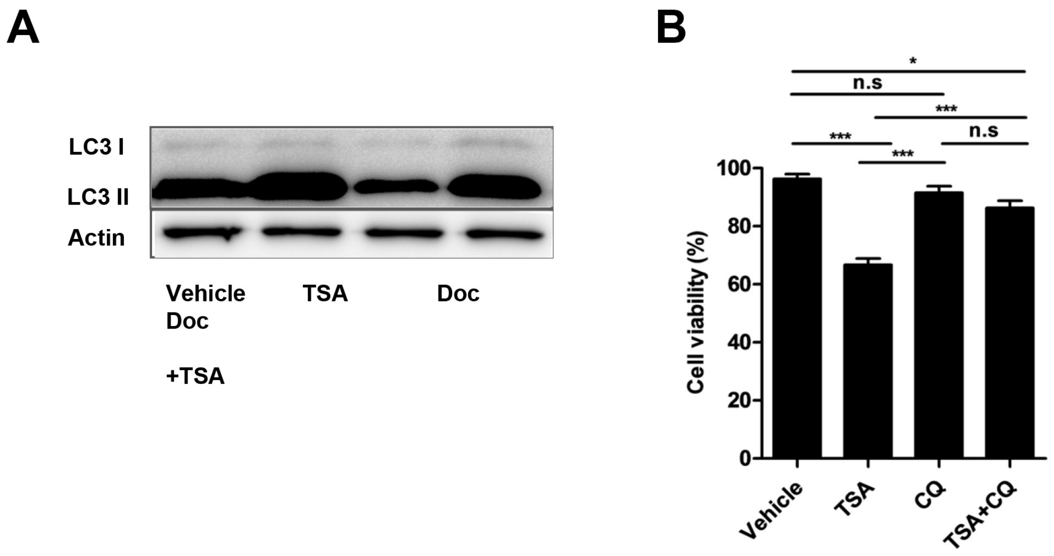


**Supplementary Figure 5: TSA-induced autophagy activation and apoptotic cell death.** **(A)** Her2/CT26 cells were treated TSA or both docetaxel (Doc) and TSA. The conversion of LC3-I to LC3-II was assessed using Western blots. **(B)** Her2/CT26 cells were treated TSA or both chloroquine (CQ) and TSA. Cell viability was evaluated at 24 h after treatment and was determined based on the absorbance at 480 nm. (*$p<0.05$, **$p<0.01$, and***$p<0.0001$, ns; not significant, ANOVA).
